# Analysis of severe adverse effects following community-based ivermectin treatment in the Democratic Republic of Congo

**DOI:** 10.1186/s40360-019-0327-5

**Published:** 2019-08-16

**Authors:** Jean-Claude Makenga Bof, Daniel Muteba, Paul Mansiangi, Félicien Ilunga-Ilunga, Yves Coppieters

**Affiliations:** 1 School of Public Health, Université Libre de Bruxelles (ULB), Route de Lennik numéro 808 à 1070, Brussels, Belgium; 2 National Program for Onchocerciasis Control (NPOC), Kinshasa, Gombe Democratic Republic of Congo; 3 School of Public Health, Faculty of Medicine, Université de Kinshasa (UNIKIN), Route de Kimwenza, Lemba Kinshasa, Democratic Republic of Congo; 4 Institut Supérieur des Techniques Médicales (ISTM), Route de Kimwenza, Lemba Kinshasa, Democratic Republic of Congo

**Keywords:** Onchocerciasis, Ivermectin, Severe adverse effects, Communities, Community-directed treatment with ivermectin, Democratic Republic of Congo

## Abstract

**Background:**

The progress of mass, community-directed, treatment with ivermectin (CDTI) for onchocerciasis control was disrupted by severe adverse effects (SAE) in the Democratic Republic of Congo (DRC). The study aimed at determining the frequency of post-CDTI SAE as well as factors associated with the occurrence of SAE.

**Methods:**

Our retrospective study relied on SAE collection cards, as archived by the DRC Ministry of Health, and compiled for people who benefited from ivermectin treatment then further developed SAE. The study included 945 post-CDTI SAE recorded in DRC between 2003 and 2017. These cases occurred in 15 projects out of 22 projects implemented in the country. All cards were reviewed and analysed.

**Results:**

Between the years 2003 and 2017, the total average population treated was around 15,552,588 among which 945 cases of SAE were registered in DR Congo, i.e. 6 cases of SAE for 100,000 persons treated per year. 55 deaths related to post-CDTI SAE were recorded, which represents 5.8% of all cases of SAE. Non-neurological SAE were dominated by severe headaches (74.8%), myalgia (64.0%) and arthralgia (62.7%). Neurological SAE were mainly coma (94.1%), motor deficit (75.4%) and palpebral subconjunctival haemorrhages (38.8%). Factors associated with the occurrence of SAE were: male, age over 18 years old, alcohol consumption, hemp intake and the presence of loiasis. The study also highlighted weaknesses of the National Program for Onchocerciasis Control (NPOC)  in terms of awareness campaigns among the population.

**Conclusion:**

Co-endemicity of loiasis and onchocerciasis is one of the key factors responsible for the occurrence of SAE following ivermectin treatment. Mobilization of resources necessary to the appropriate management of SAE and awareness of populations are essential to achieve onchocerciasis control in DRC.

## Background

Human onchocerciasis is a parasitic disease caused by *Onchocerca volvulus*, transmitted by *Simulium* females [1, 2]. Onchocerciasis causes several clinical signs such as itching, nodules, dermatosis, depigmentation and blindness in the final stage [2–4]. In socio-economic terms, it leads to the loss of employment and social status (rejection and stigmatization) [5, 6]. School absenteeism is another consequence, as, when a family member is sick, children often become “blind guides” [7]. Human onchocerciasis is considered as a tropical neglected disease affecting poor populations living in tropical and subtropical regions [8]. The infection would affect, according to estimations, more than 37 million people worldwide; 90 million people at risk live on the African continent [9]. Its endemic area crosses the whole African continent and reaches south-western Asia. Infection outbreaks were also recorded in Yemen, Oman and Saudi Arabia. On a smaller scale, outbreaks were reported in Ecuador, Venezuela, Colombia, southern Mexico and Guatemala [10, 11]. According to the World Health Organisation (WHO), 99% of people infected worldwide live in 31 sub-Saharan countries [12, 13]. In these areas, onchocerciasis is the second cause of avoidable blindness in approximately 270,000 people [13]. It is responsible for visual disorders in almost 500,000 people [13].

In the Democratic Republic of Congo (DRC), onchocerciasis is a serious public health problem due to its endemic status in all provinces [14]. More than 38 million people would be at risk of infection, which means 41% of the whole population. About 70,000 people, i.e. one in a thousand inhabitants, suffer from blindness [15]. The DRC is the country most affected after Nigeria, although onchocerciasis-related blindness is more often recorded in DRC [16]. Current efforts aiming at controlling onchocerciasis are built around the strategy of mass, community-directed, treatment with ivermectin (CDTI). It was elaborated by the African Program for Onchocerciasis Control (APOC) and implemented in each country under the form of National Programs for Onchocerciasis Control (NPOC). The strategy involves communities in the process of disease control, by building confidence and partnership between care services and communities and through the reinforcement of national health systems [17, 18]. The CDTI is a program in which communities are responsible for mass ivermectin treatment of the population [19]. Ivermectin, i.e. Mectizan® (Merck Sharp & Dohme Chibret Laboratory), is used to treat onchocerciasis. It is administered orally and eliminated in about 72 h. Its half-life is approximately 12 h [20]. To be effective, a single dose of 150–200 per kg of body weight must be taken annually. The dose depends on the person’s body mass, which can be estimated through his/her height [21]. In 1987, the Merck & Co. Inc. Company, which manufactures ivermectin, announced that it would provide ivermectin for free as long as it would be necessary [21]. Such unprecedented donation is managed by the Mectizan donation program, which works in close collaboration with Ministries of Health and other national partners [21]. The use of ivermectin has radically modified treatment conditions of filariasis over the past 30 years [22]. Its efficacy and good tolerance were assessed in the case of onchocerciasis, leading to the implementation of large-scale treatment programs. The particularity of CDTI extension in forest areas is that onchocerciasis is co-endemic with loiasis, which generates the occurrence of severe adverse effects (SAE). Besides, the first studies have proven that ivermectin was well tolerated, even in patients with high parasitic load [23–28]. When faced with such promising results, large-scale treatments of onchocerciasis were also implemented in areas of co-endemicity with loiasis. In the wake of mass campaigns led in forest areas of Cameroon, only seven cases of severe impairments were registered in the days following ivermectin treatment, since 1991 [29–33]. In DRC, the first ivermectin treatments led in savannah areas (year 2000) only generated minor (MiAE) and moderate (MoAE) adverse effects [34]. However, the first cases of SAE occurred in 2003, at the time treatments started in forest areas. Several studies, both in a hospital environment and in the field, allowed identifying loiasis as the main risk factor for developing SAE [35].

While the World Health Organisation (WHO) aims at eradicating onchocerciasis by 2025, it is clear that the disease is still far from being eradicated in DRC, after 15 cycles of CDTI [36]. Some health zones were not treated due to the social and political insecurity and by SAE observed during CDTI, which hindered the local population from continuing the treatment. The WHO recommends an 80%-CDTI treatment coverage and a 100%-geographical coverage [37] However, these conditions were not fulfilled in DRC for a complete cycle [37].

## Methods

### Study objective

The severity of SAE prompted us to perform the present study whose objective was to determine the frequency of SAE post-ivermectin treatment in DRC, as well as factors associated with the occurrence of SAE.

### Study setting

**Fig. 1:**
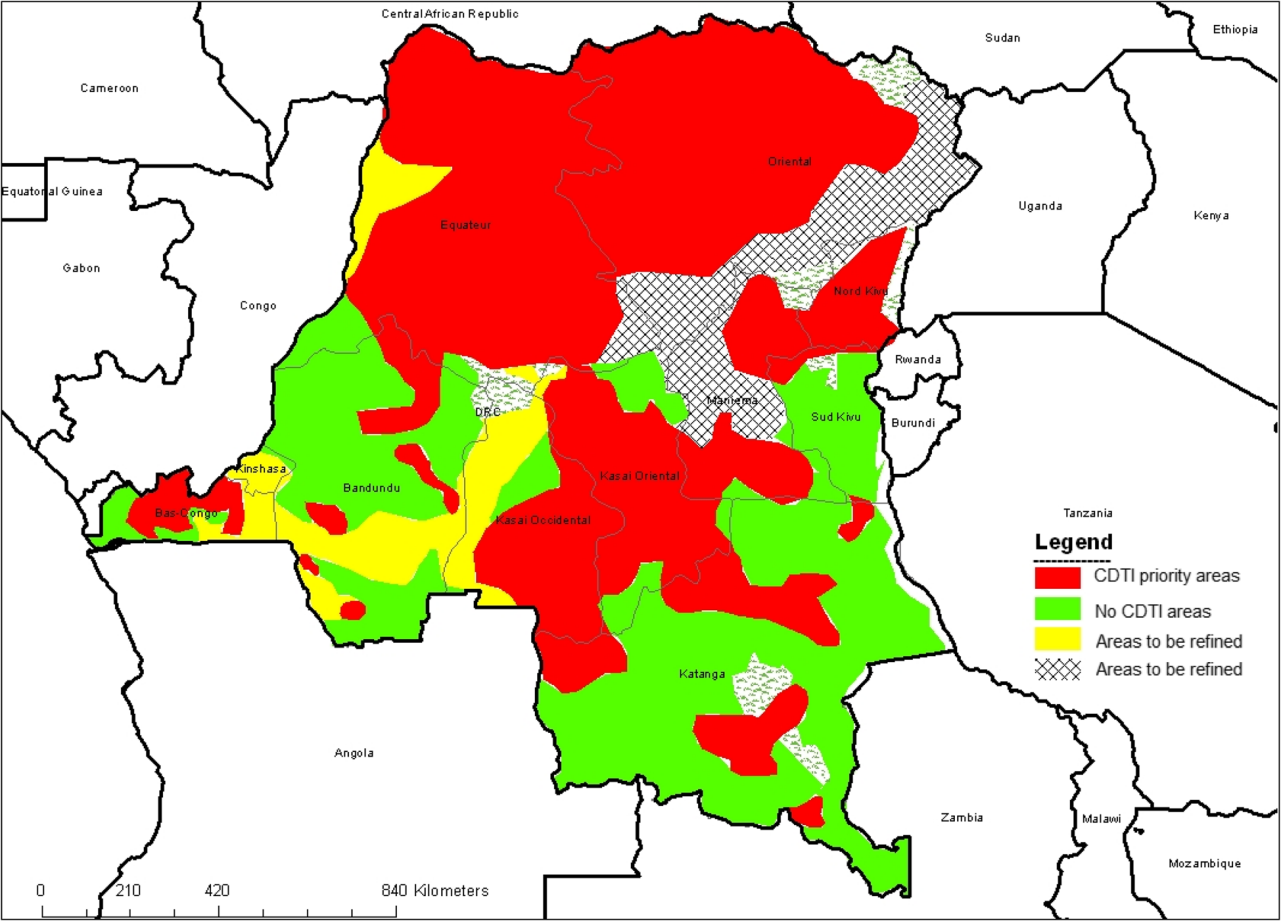
Map of CDTI areas in DRC. Source: [38]

The DRC is a Central African country with an area of 2345 million square kilometers. There are two seasons, i.e. dry and rainy. Seasons are not evenly distributed over the whole territory and their duration is variable. In the northern part of the country, rainy seasons last from April to the end of June and from September to the end of October. Congolese forests are extended between 03° north and 04° south latitude, in a region where annual rainfall reaches at least 1000 mm per year. They cover half of the territory, i.e. 125 million ha. The grassy savannah appears as one moves away from the forest and equator, to the northeast and southeast; savannah is replaced by bush and vegetation becomes clearer. Mountain vegetation evolves in successive stages as one climbs to the top: forest, savannah, bamboos, shrubs, grasses then 4000 m-vegetation. In DRC, mountains are bipolar, i.e. they are found in the southwest (Bas Congo and locally in Kinshasa [Mount Mangengenge]) and in the east of the country. Bioecological conditions and the country river system are an optima environment for the development of onchocerciasis vector, i.e. Simulium damnosum (Cf. Fig. 1: Map of CDTI areas in DRC).

### Characteristics of participants

This retrospective study relied on SAE collection cards, as archived by the Ministry of Health, and compiled for people who benefited from ivermectin treatment then further developed SAE. A total of 945 patients, from 15 out of the 22 CDTI projects implemented in DRC, were included in the study over the 2003–2017 period.

### Data collection

The data were collected by medical doctors from various hospitals in the DRC, who were previously trained for this purpose by the Ministry of Public Health. Collection cards compiling all data on people who developed SAE were made available by the Ministry of Health and further analysed. The recording of SAE cases is part of the National Program for the Control of Neglected Tropical Diseases (NPCNTDs). The following information was collected: (1) patient’s data: age and sex; (2) patient’s health status before ivermectin treatment: parasitic infestations such as onchocerciasis, loiasis, lymphatic filariasis, schistosomiasis, and palpebral subconjunctival haemorrhages (PSCH); (3) information on the last treatment: dosages of Mectizan®, praziquantel and albendazole, alcohol consumption, hemp intake and first treatment; (4) description of SAE; (5) patient’s condition at the last medical examination: hospitalisation, treatment received, rehydration, symptomatic treatment, diet, care, motion to prevent bed sores, complementary examination, complete recovery/death, duration of hospitalisation, and discharge.

### Definitions of key concepts

Adverse drug reaction (ADR) is defined as a noxious and unintended reaction which occurs after ivermectin intake, at doses normally used on a man for the prophylaxis, diagnosis or treatment of a disease or modification of a physiological function (WHO, 1972). The description concerns the patient’s response, thus individual factors might play an important role. It is a noxious phenomenon (for example, an unexpected therapeutic response can be a secondary effect but not unintended) [34].

Minor side effects (MiSE): these reactions occur after ivermectin intake and generally disappear spontaneously after 2 to 3 days. They include: fever, headaches, myalgia, arthralgia, localised or generalised oedema, asthenia, vertigos, itching, skin rash (urticaria) and blurred vision [34].

Moderate side effects (MoSE): side effects requiring care in addition to treatment disruption. In general, a side effect is characterised as moderate if, without being “serious”, it can lead the patient to seek medical attention at the health centre or to interrupt temporarily its activities. Intense headaches, generalised pain, diarrhoea, dehydration, extreme physical asthenia, blurred vision and extended immobilisation fall in this category [34].

Severe adverse effects (SAE): an adverse effect behind the death, the threat to the patient’s life at the time the event occurs, the need to hospitalise or to extend the duration of hospitalisation, after-effects or significant and lasting disability (disability means any failure to achieve daily gestures), a birth defect or a peri-partum affection are severe adverse effects. Apart from the worsening of the signs mentioned above, postural hypotension and severe respiratory problems, encephalopathies most often occur on patients infested by the *Loa loa* microfilaria, in a coma and sometimes death [35].

### Statistical analyses

Quantitative data are presented as mean ± standard deviation (SD) and qualitative data are proportions. A Chi-square test or a Fisher’s exact test (for theoretical numbers below 5) was used to analyse contingency table. Raw odds ratio (OR) and their 95%-confidence interval were presented after the univariate analysis. The multivariate analysis consisted in applying a step-by-step decreasing multiple logistic regression in order to identify the factors associated with Neurological Severe Adverse Effects (NSAE). No interaction was significant. The Hosmer-Lemeshow test was used for model fitting. Any difference was considered significant if the *P* value was less than 0.05. Statistical analyses were performed in STATA® 12 software (StataCorp LLC).

## Results

The analysis of collection cards showed that the management of CDTI by community distributors, as well as the delivery of Mectizan® according to the recommended dosage, were successful. The existence of an efficient community surveillance of SAE was also noticed through a quick reference of patients to health services (average of 1.5 days after onset of SAE).

### Sample description

Between the years 2003 and 2017, the total average population at risk was estimed around 18,514,987 and the total average population treated was around 15,552,588 among which 945 cases of SAE were registered in DR Congo, i.e. 6 cases of SAE for 100,000 persons treated per year.

The mean age of patients with SAE was 38 years old, and most of them were male (70.9%). The sex ratio (male/female) was 2.4. Loiasis was confirmed in 90.7% of patients and onchocerciasis suspected in 99.1% of them. An average of 3.6 ivermectin tablets were distributed to each patient. The consumption of hemp and alcohol 24 h before ivermectin treatment was observed for 11.6 and 26.3% of patients, respectively. The median delay of SAE onset was 24 h after taking ivermectin treatment. The median duration of hospitalisation was 7 days (Table 1). Fever was observed in 75.6% of patients with SAE. All patients who developed SAE received different types of treatment: parenteral rehydration (98%), antibiotic therapy (78.4%), malaria treatment (44.4%), antipyretics (75.6%), vitamins (51.2%) and nasogastric force-feeding (11.4%). All patients benefited from nursing. During the 15-year period, DRC recorded 55 deaths due to post-ivermectin SAE, which corresponds to a 5.8%-case-fatality rate (Table 1).

**Table 1:** socio-demographic characteristics of patients who developed severe adverse effects in the Democratic Republic of Congo, over the 2003–2017 period

Parameters Category	Mean ± SD or Me (IQR)	N (%)
Age (years)	38.6 ± 15.5	
Sex		
Male		670 (70.9)
Female		275 (29.1)
Patient’s health status		
Onchocerciasis		
Confirmed		0 (0.0)
Suspected		936 (99.1)
Negative		9 (0.9)
Loiasis		
Confirmed		857 (90.7)
Suspected		30 (3.2)
Negative		58 (6.1)
Schistomiasis		
Confirmed		0 (0.0)
Suspected		3 (0.3)
Negative		942 (99.7)
Lymphatic filariasis		
Confirmed		0 (0.0)
Suspected		3 (0.3)
Negative		942 (99.7)
Information on the last treatment		
Ivermectin dosage (number of tablets per patient)	3.6 ± 0.6	
Hemp consumption 24 h before ivermectin treatment		110 (11.6)
Alcohol consumption 24 h before ivermectin treatment		206 (26.3)
Microfilaraemia (mf/ml) upon admission	1200 (440–14,400)	
Onset of adverse effects (hours)	24 (8–96)	
Duration of hospitalisation (days)	7 (5–10)	
Status at discharge		
Recovery		890 (94.2)
Death		55 (5.8)

*SD* = standard deviation, *N* = number, *IQR* = interquartile range

### Types of adverse effects and evolution under treatment

Out of the 945 patients, 631 people (66.8%) showed NSAE. For what non-NSAE are concerned, severe headaches (74.8%), myalgia (64.0%) and arthralgia (62.7%) were most often observed. Coma predominated the severe NSAE (94.1%), followed by motor deficit (75.4%) and PSCH (38.8%) (Table 2).

**Table 2:** Types of severe adverse effects registered in DRC after ivermectin treatment, between 2003 and 2017

Types of severe adverse effects	N (%)
Non-neurological SAE (*N* = 314)	
Asthenia	65 (20.7)
Vertigos	20 (6.4)
Itching	15 (4.8)
Localised or generalised oedema	9 (2.9)
Severe headache	235 (74.8)
Myalgia	201 (64.0)
Diarrhoea + vomiting	88 (28.0)
Arthralgia	197 (62.7)
Neurological SAE (*N* = 631)	
Coma	594 (94.1)
Paralysis	13 (2.1)
Palpebral SCH	245 (38.8)
Speech disabilities	188 (29.8)
Motor deficit	476 (75.4)

*SAE* = severe adverse effects, *N* = number, *SCH* = subconjunctival haemorrhage

### CDTI projects which registred severe adverse effects 2003–2017, peaked, death and onchocerciasis and loiasis co-endemicity in the DRC

**Fig. 2:**
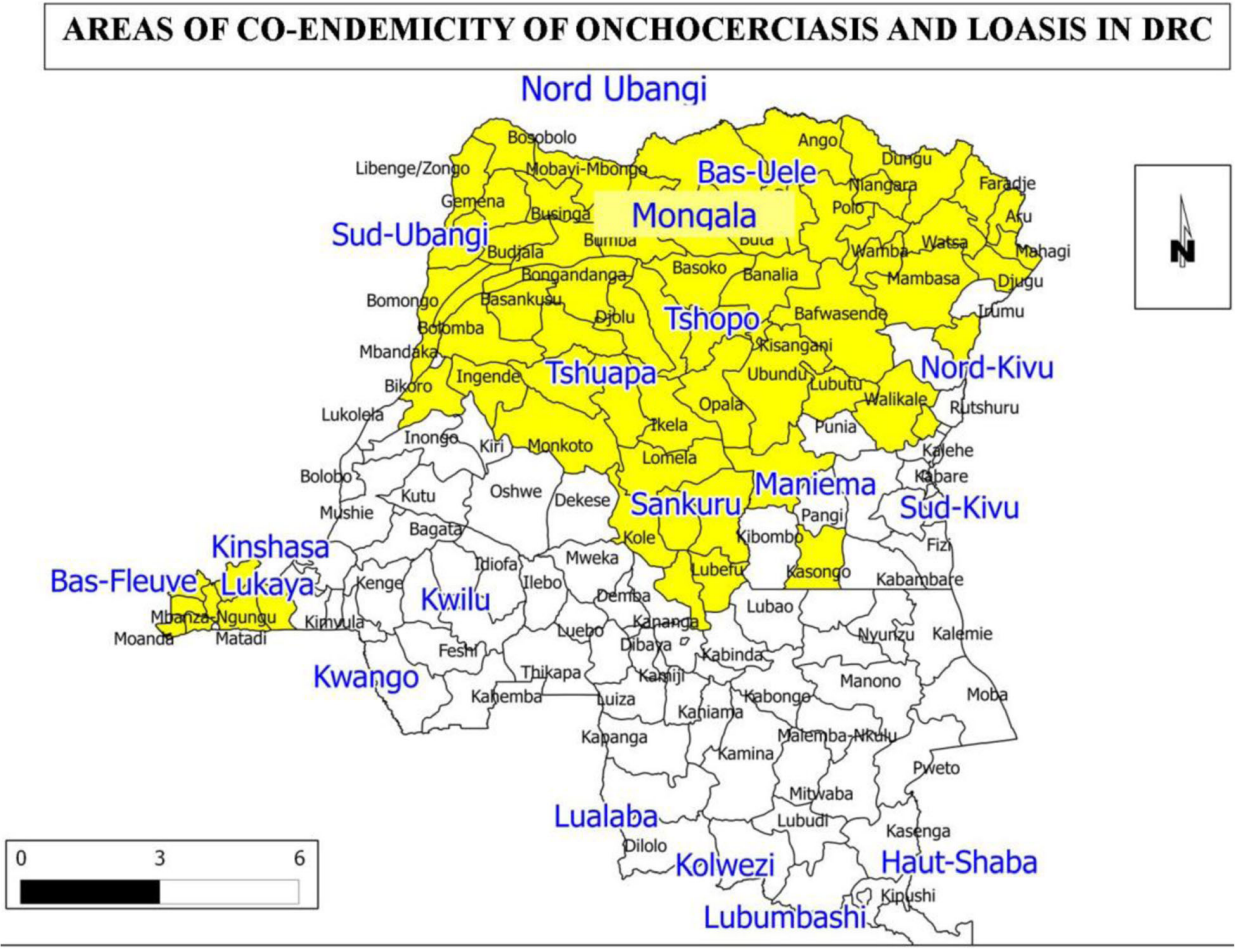
Mapping of onchocerciasis and loiasis co-endemicity in DRC. Source: [39]

At present, out of the 22 CDTI projects implemented in DRC, 15 of them faced the occurrence of SAE after ivermectin treatment [37]. These projects are located in Bas Congo, Beni Butembo, Equateur kiri, Ituri nord, Ituri Sud, Kasongo, Lubutu, Masisi Walikale, Mongala, Nord Ubangi, Sankuru, Sud Ubangi, Tshuapa,Tshopo and Uélés (Cf.Fig. 2: Mapping of onchocerciasis and loiasis co-endemicity in DRC). This map shows the CDTI projects which registered severe adverse effects after ivermectin treatment between 2003 and 2017 in the DRC).

**Fig. 3:**
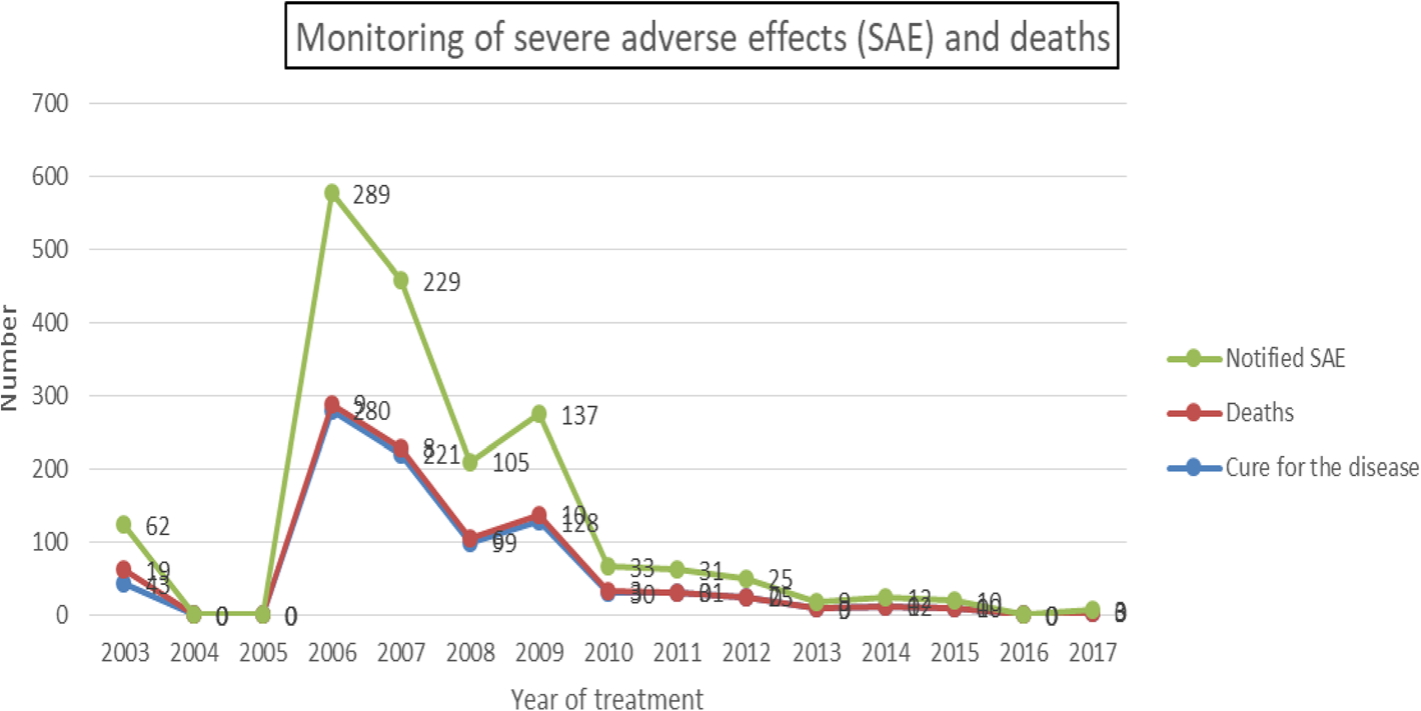
Monitoring of severe adverse effects and deaths after ivermectin treatment in the DRC, between 2003 and 2017

Severe adverse effects peaked in 2006, 2007 and 2008, while more deaths were registered in 2006 (Cf. Fig. 3: Monitoring of severe adverse effects and deaths after ivermectin treatment in the DRC, between 2003 and 2017).

From 2004 to 2005, all CDTI activities implemented in DRC were interrupted due to the occurrence of the first SAE in Bas Congo and Tshopo [14]. Onchocerciasis and loiasis epidemiological situation in DRC were precisely determined through the “Rapid Assessment Procedure for Loiasis” (RAPLOA) and the “Rapid Epidemiological Assessment” for Onchocerciasis (REA) [14] (Fig. 2): Mapping of onchocerciasis and loiasis co-endemicity in DRC.

### Factors associated with neurological severe adverse effects – univariate and multivariate analyses

The univariate analysis revealed that the risk of developing NSAE was 3.9 times higher on male patients and more important for patients over 18 years of age (10 times and 7 times higher for people aged between 19 and 35 years old and those over 35 years of age, respectively). The risk for NSAE was, respectively, 4.6 and 2.3 times more important for patients who had consumed alcohol and hemp 24 h prior to ivermectin treatment (Table 3). However, the risk of NSAE was not significantly influenced by pathologies such as malaria or diabetes (the risk of SAE occurrence was even higher for people not suffering from malaria or diabetes), except the co-infection with loiasis increased by 2.5 times the risk of SAE occurrence compared to patients who were infected by *Loa loa*. (Table 3). After fitting for age, sex, alcohol consumption, hemp consumption and associated pathologies, the multiple logistic regression highlighted several factors independently associated with NSAE: being a male, an age over 18 years old, alcohol or hemp consumption 24 h prior to ivermectin treatment and a concomitant infection with *Loa loa.* The co-infection with loiasis increased by 3 times the risk of SAE occurrence compared to patients who were infected by *Loa loa.* (Table 4).

**Table 3:** Factors associated with the occurrence of neurological severe adverse effects following ivermectin treatment in DRC between 2003 and 2017 – univariate analysis

Parameter category	% NSAE	Crude OR (95%-CI)	*P*-value
Sex			
Male	509 (76.0)	3.9 (2.9–5.4)	< 0.001
Female	122 (44.4)	1	
Age (years)			
≤ 18	14 (22.2)	1	
19–35	301 (74.7)	10,3 (5.5–19.5)	< 0.001
≥ 36	316 (65.9)	6.8 (3.6–12.7)	< 0.001
Alcohol consumption 24 h before ivermectin treatment			
Yes	181 (87.9)	4.6 (2.9–7.6)	< 0.001
No	450 (60.9)	1	
Hemp consumption 24 h before ivermectin treatment			
Yes	89 (80.9)	2.3 (1.4–3.9)	< 0.001
No	542 (64.9)	1	
Associated diseases			
Loiasis			
Yes	590 (68.8)	2.5 (1.6–3.9)	< 0.001
No	41 (46.6)	1	
Uncomplicated malaria			
Yes	610 (66.7)	1	
No	21 (70.0)	1.2 (0.5–2.6)	0.703
Diabetes			
Yes	12 (100.0)	NA	
No	619 (66.4)		

*NSAE* = neurological severe adverse effects, *OR* = odds ratio, *CI* = confidence interval

**Table 4:** Factors associated with the occurrence of neurological severe adverse effects following ivermectin treatment in DRC between 2003 and 2017 – multivariate analysis

Parameter category	Adjusted OR (95% CI)	*P*-value
Sex		
Male	1.9 (1.4–2.7)	
Female	1	< 0.001
Age (years)		
≤ 18	1	
19–35	15.0 (7.7–29.1)	< 0.001
≥ 36	8.1 (4.2–15.4)	< 0.001
Alcohol consumption 24 h before ivermectin treatment		
Yes	2.8 (2.1–3.8)	< 0.001
No	1	
Hemp consumption 24 h before ivermectin treatment		
Yes	2.3 (1.4–3.9)	< 0.001
No	1	
Associated diseases		
Loiasis		
Yes	3.4 (2.1–5.5)	< 0.001
No	1	

*OR* = Odds ratio, *CI* = confidence interval

## Discussion

Our study aimed at estimating the frequency of post-CDTI SAE and factors associated with their occurrence. During the 15 cycle-period, i.e. between 2003 and 2017, 945 cases of SAE were recorded, among which 631 (66.8%) developed NSAE. Loaisis was confirmed in 90.7% of patients and onchocerciasis suspected in 99.1% of them. Furthermore, loiasis infection increased by 3 times the risk of SAE occurrence.

Our results confirmed the findings of Ducorps et al. who have reported 112 post-ivermectin SAE cases in Cameroon since 1991 [22]. As already shown by Kamgno et al., post-ivemectin NSAE can occur in patients carrying a high *Loa loa* microfilaraemia load [36]. Our results are also in agreement with Chippaux et al. who highlighted the unacceptability of a large-scale ivermectin treatment in an area where onchocerciasis and loiasis are co-endemic due to the high risk of SAE [40]. There was an interruption of the CDTI between 2004 and 2005 because of the occurrence of SAE in coendemic areas. Although a recovery of the CDTI after this interruption, a peak of SAE occurred in 2006, 2007 and 2008. The most important peak of SAE were observed in 2006 following the occurrence of many cases of death. Our observation is different from those of Kamgno et al. who argue that despite over 350 million people being safely treated with ivermectin in endemic or co-endemic areas, there have been rare cases of death post-CDTI; these cases are most often associated with high Loa loa microfilaremia [41]. Wanji et al. also mentioned that persons with a high *Loa loa* microfilaraemia load were at risk of developing SAE after ivermectin treatment [42]. Severe adverse effects are thus a potential obstacle to advance the CDTI process, and even for onchocerciasis eradication. Therefore, we strongly encourage research on SAE pathogenesis and experimental studies in DRC, as suggested by Wanji et al. [42]. They should allow a better control of onchocerciasis and loiasis co-endemicity and thus lead to eradicate both diseases in DRC.

Our results showed that coma was the predominating NSAE (94.1%). Nzolo et al. had shown previously that the most frequent neurological trouble observed on patients suffering from *Loa loa* encephalopathy, temporally related to ivermectin intake, was also coma (74%) [43]. Out of the 631 NSAE recorded in our study, 245 patients (38.8%) had developed PSCH after ivermectin administration. The same SAE, i.e. PSCH, had previously been identified on 41 patients with NSAE by Fobi et al. after examining 1682 patients [44].

Several other post-ivermectin SAE were also observed in our study, such as itching (4.8%), localised or generalised oedema (2.9%) and asthenia (20.7%). Similar SAE had been observed in Nigeria on 38% of patients, i.e. itching (18.5%), face and limb oedema (8.2%) and asthenia (2.4%) [45].

Our results showed that an acute consumption of alcohol was a main factor for SAE occurrence, which is contrary to the results of Homeida et al. who highlighted the non- influence of alcoholic beverages [46]. Hemp (cannabis) was independently associated with the development of NSAE, which confirms the findings of Sparsa et al.; they demonstrated the occurrence of sinus tachycardia with asthma-like dyspnoea following ivermectin administration [47]. Grotenhermen strongly discourage the consumption of hemp (cannabis) due to its interaction with medicine affecting cardiac function [48]. Cannabis is thus a factor associated with the development of NSAE.

The occurrence of SAE, and associated deaths (5.8%) negatively impacted the community participation to the CDTI in DRC, thus hindering the NPOC to reach the objectives of 80%-therapeutic and 100%-geographic coverage as recommended by WHO to achieve disease eradication [49]. Makenga et al. drew attention to the lack of awareness and mobilisation of population regarding onchocerciasis, ivermectin and SAE. Accordingly, treatment discontinuation or refusal and the fear of taking the medicine due to SAE were observed in the DRC community [37]. The potential of using the LoaScope-based test - and- not - treat strategy in DR Congo in areas where deaths have disturbed the CDTI is essential in order to be able to quickly identify persons at risk of SAE and to treat them effectively as well. In their study, Kamgno and al confirm that the LoaScope-based test-and-not-treat strategy enabled the reimplementation of community-wide ivermectin distribution in a heretofore “off limits” health district in Cameroon and is a potentially practical approach to larger-scale ivermectin treatment for lymphatic filariasis and onchocerciasis in areas where L. loa infection is endemic [50]**.** On the other hand, in Nigeria (in Kwara particularly), after a strong mobilisation of the population and efficient awareness campaign, the occurrence of SAE had no effect on the community participation to the CDTI, as demonstrated by Oyibo and Fagbenro [49]. The DRC should learn from Nigeria and implement awareness campaigns. Indeed, the success of CDTI relies on the active and continuing participation of the community if one hopes to control the disease someday.

Our study was retrospective and did not consider the effect of dose-response for the quantity of alcohol and/or hemp (cannabis) according to SAE occurred. Based on WHO’s publication on the settling of alcohol-related problems, it has been pointed out that alcohol interacts with many drugs according to the quantity consumed. Therefore alcohol may change or alter the reaction of a drug [51].

On the other hand, our study is limited to demonstrate the association between hemp (cannabis) and SAE occurred. Our study did not consider as well the harmful effects of hemp (cannabis), which can also have an influence on the occurrence of the SAE. Indeed, Grotenhermen and Kirsten Müller showed that the most common cannabinoids side’s effects are tiredness, dizziness, psychological effects and a dry mouth, all of these side effects are similar to those that occurred after taking ivermectin; so they may skew determination of the SAE [52].

Our study focused only on the factors associated with NSAE. Indeed the occurrence of these effects reduced community adherence to treatment as demonstrated by Makenga et al. in their study related on untreated villages and factors associated with the absence of CDTI in DRC.

A prospective study should have highlight the relationship between the quantity of alcohol consumed and or hemp (cannabis) used with the occurrence of the SAE.

## Conclusion

The occurrence of SAE following treatment of onchocerciasis with ivermectin is clearly assessed in DRC, essentially in areas where onchoceraisis and loiasis are co-endemic. Some factors such as being a male, being over 18 years of age, alcohol consumption and hemp (cannabis) intake 24 h before ivermectin administration were associated with the occurrence of SAE. A strong mobilisation and awareness of populations, along with an adequate management of SAE, are required to control onchocerciasis.

## Data Availability

“The dataset(s) supporting the conclusions of this article is (are) included in the article (and its additional file(s): Tables and figures)”.

## Abbreviations

ADR: Adverse Drug Reaction
APOC: African Program for Onchocerciasis Control
CDTI: Community Directed Treatment with Ivermectin
DRC: Democratic Republic of Congo
MiAE: Minor Adverse Effects
MiSE: Minor Side Effects
MoAE: Moderate Adverse Effects
MoSE: Moderate Side Effects
NPCNTD: National Program for Neglected Tropical Diseases Control
NPOC: National Program for Onchocerciasis Control
NSAE: Neurological Severe Adverse Effects
OR: Odds Ratio
PSCH: Palpebral SubConjunctival Haemorrhages
RAPLOA: Rapid Assessment Procedure for Loiasis
REA: Rapid Epidemiological Assessment for Onchocerciasis
SAE: Severe Adverse Effects
SD: Standard Deviation
ULB: Université Libre de Bruxelles
WHO: World Health Organization
